# Self-management support (SMS) in primary care practice: a qualitative focus group study of care professionals’ experiences

**DOI:** 10.1186/s12875-024-02317-4

**Published:** 2024-03-01

**Authors:** Lotte Timmermans, Dagje Boeykens, Muhammed Mustafa Sirimsi, Dominique Van de Velde, Patricia De Vriendt, Peter Decat, Veerle Foulon, Ann Van Hecke, Mieke Vermandere, Birgitte Schoenmakers, the Primary Care Academy

**Affiliations:** 1 Academic Centre of General Practice, KU Leuven, Kapucijnenvoer 7, Box 7001, 3000 Leuven, Belgium; 2 Department of Rehabilitation Sciences, Ghent University, Ghent, Belgium; 3 Department of Public Health and Primary Care, Ghent University, Ghent, Belgium; 4 Centre for Research and Innovation in Care, University of Antwerp, Antwerp, Belgium; 5 Department of Rehabilitation Sciences, Occupational Therapy, Faculty of Medicine and Health Sciences, Ghent University, Ghent, Belgium; 6 Department of Occupational Therapy, Artevelde University of Applied Sciences, Ghent, Belgium; 7 Frailty in Ageing (FRIA) Research Group, Department of Gerontology and Mental Health and Wellbeing (MENT) Research Group, Faculty of Medicine and Pharmacy, Vrije Universiteit, Brussels, Belgium; 8 General Practice and Primary Health Care, Ghent University, Ghent, Belgium; 9 Clinical Pharmacology and Pharmacotherapy, KU Leuven, Leuven, Belgium; 10 University Centre for Nursing and Midwifery, Ghent University, Ghent, Belgium; 11 Department Nursing Director, Ghent University Hospital, Ghent, Belgium

**Keywords:** Self-management support, Healthcare professionals, Primary health care, Qualitative research

## Abstract

**Background:**

To support self-management of chronically ill persons, innovative approaches of care practice are being developed. Unfortunately, many self-management supporting interventions struggle to achieve reliable and consistent improvements at various levels (patient, provider and healthcare system level). One possible strategy to facilitate translating theory into practice, is to consider the healthcare professionals’ perspective prior to the development of new interventions. An exploration of their knowledge and opinion about barriers and facilitators is necessary before employing any self-management support (SMS) intervention. Therefore, our study aims to explore care professionals’ perspectives about SMS within the Flemish primary care setting.

**Methods:**

This study used a qualitative study design to examine SMS in primary care setting. Five focus groups were conducted, grouped into three waves. Participants were healthcare professionals in Flanders representing different disciplines and settings. A maximum variation purposive sampling was used to recruit participants. For the data analysis, the framework of thematic networks by Attride-Stirling was applied.

**Results:**

A total of 34 healthcare professionals participated. Three global themes related to SMS were derived from the thematic analysis: (1) Characteristics, (2) Support strategies, (3) Barriers and facilitators. SMS was characterised as a collaboration-based and person-centred approach. A variety of supporting strategies were mentioned by the focus group participants. Most strategies consisted of informing and educating patients. Complementary to individual strategies, collaborative strategies were deemed necessary to support self-management. Regarding barriers and facilitators, different patient-related factors were identified. Additionally, competencies of healthcare providers and external factors seem to hinder the implementation of SMS in practice.

**Conclusions:**

This focus group study highlights the importance of a collaborative, person-centred approach to SMS in the context of chronic diseases. Our findings point to the need for interventions that raise awareness and address barriers associated with SMS. Since generic SMS does not exist, the road to success is a growth process in which support must be adapted to the individual patient.

**Supplementary Information:**

The online version contains supplementary material available at 10.1186/s12875-024-02317-4.

## Background

The world’s population is undergoing a demographic shift. Over 70% of European citizens will be over 65 years old by 2050 [1]. This poses many challenges to our healthcare system. The evolution will result in an increasing number of people living with a chronic condition. At this moment, chronic non-communicable diseases (NCDs) are among the most prevalent causes of mortality and morbidity in Europe [2]. In addition, chronic diseases substantially contribute to the global burden of disease, with cardiovascular disease, cancer, type 2 diabetes, and chronic respiratory disease being the most common ones [3, 4].

Various approaches are adopted to prevent diseases, improve diagnostics, and provide more effective therapies [1]. In addition, the focus is on patients’ involvement and participation in the care process. When provision of care becomes patient-centred, patients feel empowered and become actively involved in their care process [5]. Moreover, activating and involving patients contributes to developing patients’ self-management strategies [6] and results in improved health outcomes [7]. The concept of self-management has been extensively explored in the literature [8]. Barlow et al. (2002) defined self-management as “the individual’s ability to manage the symptoms, treatment, physical and psychosocial consequences and lifestyle changes inherent in living with a chronic condition” [9]. According to literature, self-management could positively influence health service utilization, financial costs, and clinical outcomes (including reduced morbidity and mortality) [10–12]. Moreover, a positive impact on various patient-related outcomes (including patients’ self-efficacy and activation) and quality of life outcomes is reported.

The ability to self-manage chronic diseases requires six skills that patients should acquire: action planning, decision making, development of a patient–provider partnership, problem solving, self-tailoring, and utilization of resources [13]. Various care interventions focus on the further development of these skills in chronically ill patients. Hence, further development and support of self-management skills are a prerequisite for achieving desired health and quality of life outcomes. These skills are not innate and do result from a lifelong learning process and task [8]. Involving patients’ care team, and fundamentally, the primary care professional, plays a crucial role in developing self-management skills [14, 15]. More specifically, healthcare professionals can encourage and support patients to develop skills and knowledge needed to manage their condition effectively, and thus engage in self-management [16]. Furthermore, healthcare professionals can assist patients in setting goals and developing personalized care plans [17, 18]. By collaborating with patients, healthcare professionals can help optimise self-management support (SMS) and contribute to improved health and quality of life outcomes. Therefore, the holistic definition of SMS by Adams et al*.* (2004), which defines SMS as “the systematic provision of education and supportive interventions by health care staff to increase patients’ skills and confidence in managing their health problems, including regular assessment of progress and problems, goal setting, and problem-solving support”, was used for this study [19].

When implementing SMS approaches, different internal and external factors are taken into account to achieve effective and sustainable interventions tailored to the individual patient’s needs and preferences [20–22]. Nevertheless, many interventions struggle achieving reliable and consistent improvements in care practice [23]. Often, these interventions are designed with only the patient’s perspective in mind, without considering the perspectives of the care professionals who have to implement and support these SMS interventions [24]. This oversight has led to a notable gap in our understanding, as the experiences, challenges and perspectives of professionals are under-represented. To fill this gap, our study focuses on this caregiving population, shedding light on their prior knowledge and opinions about SMS. Existing literature exploring professionals’ perceptions often focuses on higher level concepts such as the provision of beneficial support or the role of professionals [25, 26]. As a result, there is a distinct lack of foundational insights from professionals at the conceptual level of SMS. In this study, we aim to uncover critical insights that have been overlooked in the current literature, ultimately contributing to a more comprehensive understanding of the factors that influence the successful implementation of SMS interventions. By exploring the experiences of care professionals, our goal is to make a significant contribution to the wider SMS puzzle. In doing so, we aim to create a synergistic relationship where the care professional perspective complements and strengthens the existing patient-centred literature. This study is part of the Primary Care Academy (PCA), a research and teaching network that aims to strengthen the capacity of primary care by developing interventions, optimal roadmaps, and hands-on toolkits for primary care policies, practice, and education, built upon the principles of goal-oriented care (GOC), interprofessional collaboration (IPC) and SMS.

## Methods

### Design

This paper reports on the results of a study conducted in Flanders, Belgium, where qualitative focus groups with healthcare professionals were carried out to explore the concept of GOC, IPC and SMS. The study contributes to the aim of the PCA and was performed by their main PhD-researchers (DB, LT and MS) under supervision of PDV and DVDV. This paper focusses on the concept of SMS. The study obtained approval from the Ethical Committee of University of Antwerp (B300201942302). The COREQ guidelines were consulted to report this focus group study [27].

### Recruitment and participants

A maximum variation purposive sampling was used to recruit the focus group participants, following the principles of Patton et al*.* (2014) [28]. The focus was on gathering insights from a wide range of healthcare professionals. More specifically, the recruitment strategy aimed a maximum variation in health and social disciplines, in academics and frontline professionals, in Flemish regions and organizations of employment in primary care via announcements of the PCA (e.g., network of the co-authors) and other healthcare organizations (e.g., the White-Yellow Cross, Flemish Patients’ Platform). Professional contacts were used to recruit participants for the focus groups by e-mail.

Five focus groups were performed in an iterative process of three waves among a purposive sample of scholars, academics, and frontline professionals respectively. Participants were included if they met the following predefined criteria. For the first wave, participants had to be a member of the PCA consortium with a certain level of expertise in one of the central concepts of GOC, IPC and SMS in primary care. Expertise refers to self-appointed theoretical and practical knowledge gained from professional experience, including research and practice. These individual experts were contacted by the main researchers and composed a heterogeneous group. For the second wave, our inclusion criteria consisted of being a frontline care professional and having knowledge because of field experiences. This wave consisted of three homogenous focus groups on GOC, IPC and SMS. Depending on participants’ expertise, they were included in one of these three parallel focus groups. For our third and last wave, the participants of the previous waves were contacted, and the final panel was heterogeneously composed based on the principles of maximum variation and the level of engagement during the earlier focus groups. The organisation of this last focus group with a mix of topics and expertise, including previous participants, was crucial for refining and validating the ideas and themes. Participants did not receive a remuneration or compensation for their participation in the study and participation was on voluntary basis.

### Data collection

Data were primarily collected through the three waves of focus groups. The first focus group took place at the start to test and finalize the interview guides. The second wave of focus groups built on the first by zooming in on one care concept in more depth. The strategy of focusing on one specific topic typically provides more information than combining multiple care topics during the same amount of time. The third wave consisted of a focus group which was organized to focus on the interactions and overlap between the three central care concepts (namely GOC, IPC and SMS). During this final focus group, findings from the earlier waves were aligned with the panel to validate our data.

Depending on the central concept(s) being covered, participants in the focus groups were asked to answer questions about the application of the care concepts in primary care practice: (1) “How do primary care professionals understand, define and describe GOC and how do they use it in practice?”; (2) “How do primary care providers understand, define and describe IPC, who is involved and how do they experience the collaboration?”; (3) “How do primary care professionals perceive SMS in primary care practice, and what barriers and facilitators affect its implementation?”. During the last focus group, the point of saturation was reached as no new ideas emerged and earlier findings were corroborated.

Focus groups were conducted in Dutch between January 2020 and September 2020 by one of the main PhD-researchers (DB, LT, MMS) of the PCA, using precomposed interview guides developed for this study. These guides were meticulously developed through iterative collaborative sessions between the researchers, incorporating evidence from practice and informed by thorough literature analysis. The specific interview guide questions related to self-management support can be found in Supplementary file 1. Dutch, the native language of the participants, was used for the focus group sessions. Before entering the focus groups, participants were asked to read and sign an informed consent form, in which they agreed to participate in the study and approved to be audiotaped. In advance, the research group received a training in qualitative research techniques to guarantee a certain level of expertise. The training covered key elements of focus group facilitation, research strategies and ethical considerations. It also provided a comprehensive exploration of different aspects of qualitative research, covering study designs, methodological approaches and analysis techniques. This training equipped the team with a diverse set of skills essential for effective qualitative research, ensuring their ability to collect nuanced and rich data during the focus group sessions. The overarching aim was to address all facets of qualitative research and enhance the team’s expertise throughout the research process.

### Data analysis

The focus groups were audio-taped and transcribed verbatim. Analysis of the data on the topic of SMS was done by the main researcher (LT), in close collaboration with the other investigators (AVH, BS, MV, PD, VF) by means of gatherings to discuss the interpretations. After drafting narrative reports of each focus group, the transcripts were coded and divided into quotations, answering our research question. For the thematic analysis of the data [29], the framework of Attride-Stirling (2001) was applied [30]. This method is characterized by a representation of the results in the form of themes. A thematic network was developed by exposing significant themes at different levels. More specifically, we identified basic themes, organizing themes and global themes. The analysing process was built on the principles of grounded theory (Corbin and Strauss, 1990) [31] and contained three different stages comprising 6 iterative steps [30].

## Results

A total of 34 participants took part in this qualitative focus group study. Table 1 presents the characteristics of the focus groups, including the professional backgrounds of the participants and their distribution across each wave.

**Table 1:** Characteristics of focus groups, organized in three waves

**First wave**			
**Focus group 1**	**Duration**	**Number of participants**	**Professional backgrounds**
*Focus group with experts of the PCA consortium*	123 min	5	Speech language pathologist Nurse Occupational therapist Pharmacist Physiotherapist
**Second wave**			
**Focus group 2a**	**Duration**	**Number of participants**	**Professional backgrounds**
*In-depth focus group with frontline professionals with knowledge of SMS*	68 min	7	Nurse (*n* = 2) Pharmacist (*n* = 2) Psychologist Social worker (*n* = 2)
**Focus group 2b**			
*In-depth focus group with frontline professionals with knowledge of IPC*	90 min	8	General practitioner Sociologist Pharmacist (*n* = 4) Psychologist Social worker
**Focus group 2c**			
*In-depth focus group with frontline professionals with knowledge of GOC*	119 min	8	General practitioner Nurse (*n* = 2) Occupational therapist (*n* = 3) Social worker (*n* = 2)
**Third wave**			
**Focus group 3**	**Duration**	**Number of participants**	**Professional backgrounds**
*Final focus group to validate with selection of previous participants*	107 min	6	General practitioner Sociologist Nurse Psychologist Social worker (*n* = 2)

*GOC* Goal-oriented Care, *IPC* Interprofessional Collaboration, *PCA* Primary Care Academy, *SMS* Self-Management-Support

After applying the framework of Attride-Stirling, three global themes related to SMS were derived from the thematic analysis: characteristics of SMS, support strategies, barriers and facilitators. We will present each theme in detail, supported by relevant quotes from the focus group discussions.

### Characteristics of SMS

**Fig. 1:**
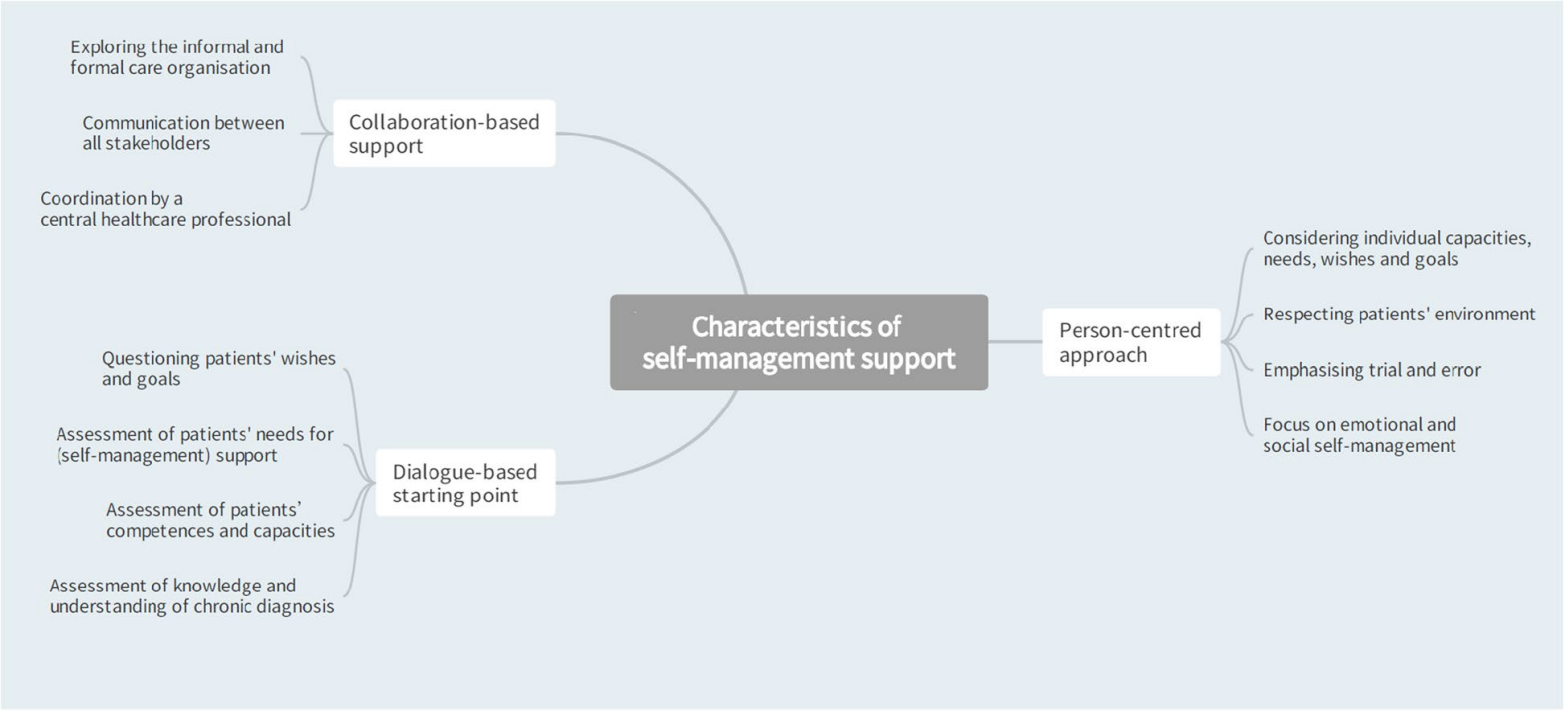
Thematic network: Characteristics of self-management support

The first organizing theme that emerged from the thematic analysis is related to characteristics of SMS (Fig. 1). Participants described SMS as a collaboration-based approach (first basic theme), which is person-centred (second basic theme) and starts based on dialogue (third basic theme).

During the focus group discussions, the question was raised whether patient care could be delivered ‘*in tandem*’ or in collaboration with the whole care team. In the care process, supporting self-management was seen as ‘*part of the journey’*. Participants stated that healthcare professionals are in charge during the care process but the responsibility for the direction of care belongs mainly to the patients themselves. Although patient-healthcare professional one-to-one interactions were strongly emphasised in the context of SMS, the support is not exclusively restricted to individual consultations. Group education, peer support and e-health were also mentioned.

“But when you start from a context of people who are chronically ill,to me that’s [SMS] really part of the journey. Where you start from a set of goals that are important to the patient, and then where you start supporting them.” (Wave 2 – Social worker).

Participants perceived supporting self-management as a dynamic, personalised process. They indicated that this process is driven by wishes, needs and goals of the individual patient, which may change over time. Therefore, healthcare professionals must engage in dialogue to explore patient’s goals as a first step towards establishing SMS. Throughout all the conversations, the importance of providing a listening ear was highlighted by the participants.

“It [self-management] is not something black, white, positive, or negative. It changes. And I think support plays a very important role in this.” (Wave 1 – Physiotherapist)

The human factor strongly emerged in the focus group discussions. Participants indicated the importance of paying attention to the patient behind the chronic disease. Healthcare professionals emphasized that a patient is ‘*a person who has received bad news and needs to deal with this news’*. According to the participants, interpersonal communication and guidance should be central to SMS. The focus should be on social and emotional management, on top of medical management. For them, there is a need to shift away from somatic and technical care as a fundamental premise in the delivery of chronic care.

“We need to be able to make that mental switch as a health care provider from not sitting there looking at a patient but looking at a person who has actually received bad news and has to deal with that.” (Wave 2 – Nurse)

### SMS strategies

**Fig. 2:**
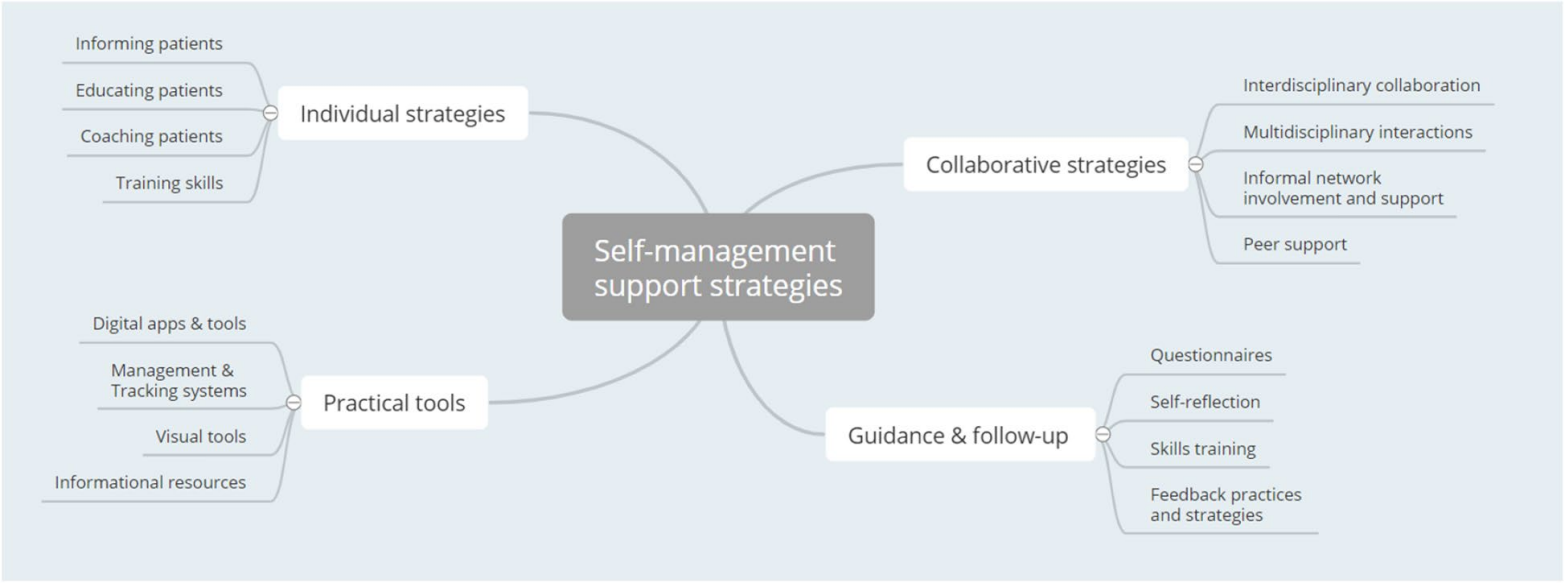
Thematic network: Self-management support strategies

The second theme that emerged from the thematic analysis is related to SMS strategies (Fig. 2). Four different types of strategies were identified: individual strategies, collaborative strategies, strategies related to guidance and follow-up, and practical tools.

The strategies discussed in the focus groups focused mainly on the direct individual support that professionals could offer to facilitate patient self-management. According to the participants, healthcare professionals were expected to function in a coaching role to support self-management. Regardless of their discipline, participants agreed that every formal care professional should learn to take on this coaching role.

“I think the role of coach … whatever care provider it is, that’s a role that we must learn to take on. And that’s different than acting and providing medical care.” (Wave 2 – Nurse)

Specific support strategies to offer one-on-one guidance in healthcare practice were mentioned. Most examples given by participants consisted of informing and educating patients. Apart from utilizing teaching and coaching methods, participants indicated that efforts should be made to train patients specific self-management skills (e.g.; managing medication, monitoring health indicators). Practicing specific skills under the supervision of a formal caregiver assures timely adjustment according to the needs and capabilities of the individual patient.

Besides individual support strategies, the strength of collaboration in supporting self-management was discussed in the focus groups. According to the participants, healthcare professionals should join forces inter- and multidisciplinary to achieve stronger support for patients’ self-management. Central actors cited included the general practitioner, social worker, nurse, pharmacist and medical specialists. In addition to the formal support network, the role of the informal network was highlighted. Participants emphasized on involving the patient’s closest environment when supporting self-management. They further stressed that the level of involvement should always be aligned with patients’ values and needs. Involving informal caregivers in the care process was pointed out as an added value in information exchange, education and skills learning. According to the participants, mapping the social network also provides insights into patients’ lives. Therefore, it was identified as an essential component of SMS.

“And I think maybe you also need to start looking, and it is not only the family or household context that needs to provide support, but just looking at who is relevant here for that person, who can be helpful here.” (Wave 2 – Social worker).

Additional to the patient’s closest environment, the importance of peers was also emphasized in the focus groups as part of informal network support. More specifically, the added value of peer support was mentioned in exchanging information on coping with chronic disease and in learning various self-management skills.

“I would like to add that patient associations also play a very important role in learning to take care of yourself, and in self-management. Through contact with peers, a lot of information can be obtained, and I think especially in addition to professional knowledge. Especially around quality of life.” (Wave 3 – Sociologist).

Since SMS is a dynamic process, participants indicated that attention should be paid to continued guidance and follow-up of patients’ self-management skills. Different follow-up strategies were discussed in the focus groups. The use of standardized questionnaires to assess self-management is one such strategy. Moreover, different self-reflection strategies were mentioned to gain insights into patients’ self-management skills and to evaluate support. In addition, several feedback strategies were discussed to question patients’ experiences regarding SMS. Finally, it was mentioned that practicing skills under supervision of a formal care provider is a low-threshold technique to evaluate patients’ progress and identify difficulties.

“I think we also have to be able to let the person that we want to help, to have them look at themselves … So, to speak, check in with themselves about how far do they think they’ve progressed …” (Wave 2 – Nurse)

Complementary to individual and group techniques to support self-management, several practical tools were discussed in the focus groups. These included digital apps and tools that can contribute to better follow-up and guidance of self-management. More specifically, practical tools to support knowledge and information sharing were discussed. For example, the use of visual techniques during medical consultation (e.g., whiteboard, drawings) were mentioned conveying information to the patient in a systematic and accessible manner. Furthermore, the importance of providing reliable sources of information (websites, medical records, leaflets or booklets) that can be consulted to supplement health appointments was highlighted.

### Barriers and facilitators to SMS

**Fig. 3:**
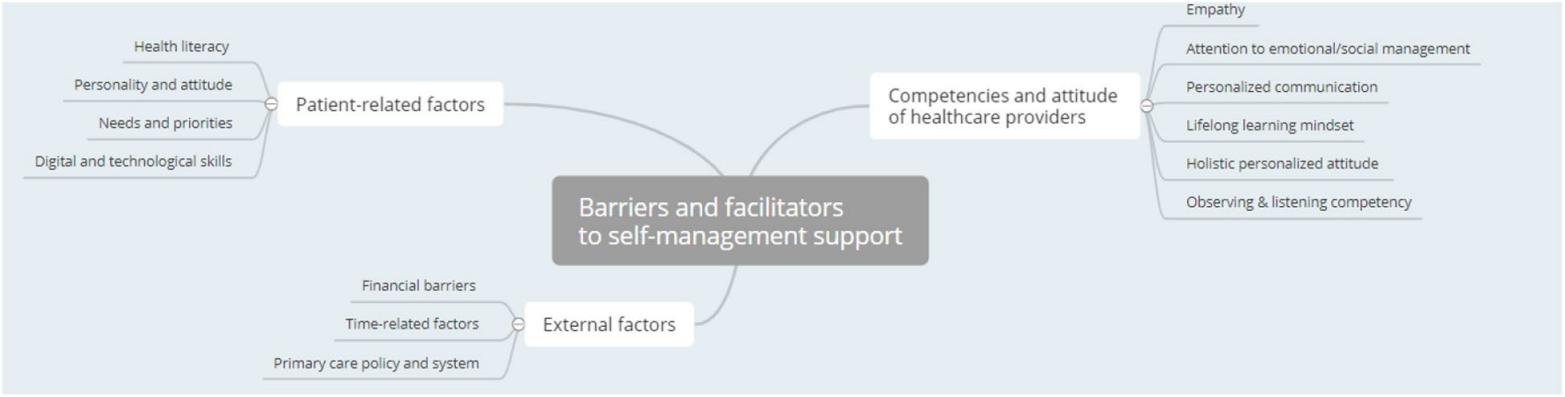
Thematic network: Barriers and facilitators to self-management support

The last theme addressed in the focus groups concerns barriers and facilitators to SMS (Fig. 3). More specifically, several factors were discussed that hamper the implementation of SMS in daily care practice. These factors were grouped into three different basic themes: patient-related factors, competencies and attitudes of healthcare providers and external factors.

According to the participants, difficulties often arise from patient-related factors. To overcome these barriers, several key elements were identified that are important for SMS. First, attention should be paid to information transfer in patients with lower health literacy. Assessing full and proper comprehension and interpretation of information was cited as an essential part of SMS.

“I think that's just very different for each patient, what they need.” (Wave 3 – Sociologist)

To facilitate understanding, the message must be tailored to the health literacy of the patients. Second, the use of modern support tools requires different digital and technical skills. Consequently, participants in the focus groups concluded that high-tech support tools are only accessible to a limited patient population.

“And of course, with a digital tool, you immediately end up with the digital divide. And the accessibility of that app, and the information around it and whether everyone can work with it.” (Wave 2 – Social worker)

Further, participants indicated that patients should be open to SMS. More specifically, specific circumstances (e.g., major life event) may contribute to different care needs and priorities. Besides time-dependent needs and priorities, participants stressed that the patient’s personality should always be considered when providing SMS. For instance, some patients prefer outcome-driven support strategies (e.g., setting personalized goals), while others have a greater need for process-oriented strategies (e.g., improving cooperation and communication in the care team). Therefore, SMS should always be tailored to the individual needs, wishes and capabilities of the patient according to the participants.

“If clients/patients are tackling problems, they need to be able to talk about them before they can be open to new ideas.” (Wave 2 – Nurse)

In addition to patient-related factors, a variety of professionals’ competencies that contribute to good SMS were mentioned. First, the role of empathy was emphasized. Healthcare professionals are expected to be able to empathize with the person’s feelings behind the patient. According to the participants, these emotions can be explored through an observing and listening attitude. They pointed out that communication is key. Second, several clinical competencies were discussed. Participants argued that these competencies make up only a limited part of SMS. More specifically, they are taught in basic education and further refined in daily care practice.

“I think tailoring communication. Sometimes using some complex words, otherwise simple words. But I think that’s a very important one.” (Wave 2 – Social worker)

“Especially listening/hearing/reading/… I do think that’s important to not fall into the trap of imposing your goals in a manipulative way.” (Wave 1 – Physiotherapist)

Participants stated that SMS extends far beyond the good use of clinical skills. The need to shift away from a purely problem-solving mindset in primary care was emphasized. To provide holistic support, attention must be paid to contextual factors affecting patient’s ability to self-manage a chronic condition. Creativity and innovative thinking empowering care professionals to tailor each patient’s unique situation could contribute to personalized support strategies. Finally, participants indicated that support is a growth process in which care providers must engage with a willingness to learn and optimize their support.

“Surely a competency could be to move away from purely problem-solving thinking. And learn not to think in somatic or technical terms, but rather yeah… have the ability to assess the mental status of the person.” (Wave 2 – Nurse).

In addition to patient and care provider-related factors, external factors contributing to SMS were also discussed. For instance, policy-related factors in primary care that hamper good support were discussed. According to the participants, becoming familiar with self-management and support strategies takes time and effort. Therefore, it was indicated that the short duration and low frequency of patient-provider encounters impedes sustained SMS. In addition, limited financial resources were also mentioned as an obstacle. Finally, participants indicated that there are still too few communication channels to support multidisciplinary SMS.

“But I think there should also be room for patients to become familiar with some tools or resources.” (Wave 1 – Speech language pathologist)

## Discussion

### Summary of results

An in-depth understanding of how professionals perceive SMS in primary care practice, and what barriers and facilitators affects its implementation is invaluable for achieving effective sustainable SMS in healthcare practice [32, 33], especially since care professionals play a crucial role in self-management [14]. Therefore, this paper aimed to explore in detail healthcare professionals’ perceptions of SMS before developing new interventions and optimising existing ones. The goal is to expand upon prior research that theoretically investigated the ingredients of effective SMS in practice [10, 34]. We learned that self-management is a collaborative, person-centred approach that starts with dialogue, and that this support does not take place exclusively in face-to-face consultations. SMS is part of a bigger picture, is a dynamic concept, making proper follow-up and temporary adjustment of care essential. SMS starts with questioning the patient’s goals and the focus should be on the person rather than the case, moving away from medical management. The role of the professional is described as that of a coach applying different supporting strategies depending on the person. Collaboration with the informal support network is considered invaluable. Finally, the use of practical tools to support self-management was discussed, as well as other barriers and facilitators to SMS. External factors such as policies and health structures were mentioned as additional challenges for SMS.

### Comparison with literature

According to the focus group participants, SMS is described as a personalised process. This is also reflected in previous research emphasising support is highly patient-dependent, since ‘the patient’ does not exist [35, 36]. In addition, professionals in the focus groups discussed that supporting self-management is a learning process. An essential starting point seems a good understanding of the concept as no uniform description emerged from the focus groups. On one hand, for example, it is suggested that healthcare professionals are ‘in charge’ of self-management. On the other hand, it is emphasized that SMS should be delivered collaboratively, in tandem, with the entire care team. Unfortunately, even in literature, the concept of self-management (support) seems ambiguous, complex and even without consensus [8, 37]. This can cause different interpretations and misconceptions among healthcare professionals. To monitor SMS, focus group participants emphasise the use of practical tools and devices. Although this can be very useful, it is important to approach it cautiously [38, 39], especially since several support techniques require digital skills from patients [40]. In addition, the use of questionnaires to guide this dynamic process is well-described in literature and was also mentioned by the focus group participants [41, 42].

When developing interventions to encourage self-management in care practice, the needs of patients, the care receivers [24, 43], must be considered in addition to the input of professionals, the caregivers [32]. According to the participants, support is ideally tailored to the goals, capabilities and needs of each patient. In a previous qualitative study, we developed a conceptual model, the SILCQ model, which describes ideal SMS from the patient perspective, based on dyadic interviews [15]. Five actions of care professionals are presented as fundamentals: the act of supporting, involving, listening, coordinating and asking questions to patients. Different parallels can be drawn when considering the voice of the professional in the focus groups. For instance, the act of supporting and coaching patients is mentioned several times by our participants. More specifically, emphasis is placed on supporting social and emotional management as part of SMS, on top of medical support. However, when discussing SMS strategies, medical approaches were primarily cited, such as learning practical techniques and practicing skills. Regarding the involving fundament in the SILCQ model, this aspect to encourage self-management similarly emerged from the focus group discussions. It was suggested that for perfect cooperation, care should be organized in tandem by providers and patients. Finally, the focus groups highlighted providing a listening ear to the patient. This is reflected in many other studies, which emphasises the importance of listening to both patients and their informal network [44, 45]. Paying attention to and engaging this informal network is an essential part of SMS [46, 47].

As noted by our participants, SMS strategies mainly consisted of informing and educating patients. The effectiveness of education has been thoroughly researched in recent years and seems promising [17, 48, 49]. Participation of peers and expert patients yielded positive outcomes in previous research [50, 51]. When discussing challenges, three main requirements for SMS were expressed in the focus groups: patient readiness, professional’ competences and a supportive health system. The latter in particular is a much-debated topic in Europe [52].

### Strengths and limitations

Some limitations should be mentioned. First, due to a high pressure on healthcare professionals generated by the COVID19-pandemic, the number of participants in the focus group discussions was rather limited. Last-minute cancellations due to illness or high workload was a remarkable barrier for conducting this study. Despite this, we were able to gather a sufficient number of participants to conduct the focus groups. It is worth noting that having more participants may not have necessarily been beneficial, as it can reduce the dynamics and effectiveness of the discussions. Second, the duration of the focus groups varied widely and different topics were combined. This may have affected the depth of discussions. However, our subsequent analysis of the data revealed a wealth of information and made us decide not to further investigate the topics among professionals after the third wave. Also, participants emphasised the value of exploring issues that are related to each other such as GOC, IPC and SMS. We strongly believe that optimising care is only possible through cooperation. One possible strategy is to consider care from a more holistic approach. An approach that transcends individual care concepts. Third, since participation in this study was completely voluntary, volunteer bias may occur. However, our research sample can be considered clinically representative with a variety of healthcare professionals included. Finally, applying the method of thematic analysis poses some challenges. The main one is the challenge of inconsistency when developing themes. However, thematic analysis provides the advantage of flexibility and promotes richness of data [53]. The Attride-Stirling thematic analysis method is strengthened by its iterative process and ongoing validation by multiple researchers, which enhances the credibility and reliability of the analysis.

### Implications for research and practice

Our study provides in-depth insights into healthcare professionals’ experiences related to SMS. To address the identified gaps and challenges, an appropriate next step is to brainstorm and develop new SMS interventions that build upon the insights gained in the focus groups. In addition, more awareness and understanding of the concept of SMS is needed, as well as the barriers and facilitators associated with it. To this purpose, interventions should be developed to raise awareness among healthcare professionals and patients and to equip them with knowledge and skills needed to apply self-management strategies effectively. The implementation of SMS can only be successful if all persons involved have a good understanding of the concepts and of what barriers and facilitators affect implementation in the intended setting.

## Conclusions

This focus group study highlights the importance of a collaborative, person-centred approach to self-management in the context of chronic diseases. Our findings point to the need for interventions that raise awareness and address barriers associated with SMS. Since generic SMS does not exist, the road to success is a growth process in which support must be adapted to patients’ individual goals, needs and capabilities. When developing new interventions, it is of utmost importance to build on the strengths of current approaches and addressing identified gaps and challenges.

### Supplementary Information

**Supplementary Material 1.**

**Supplementary Material 2.**

## Data Availability

The datasets used and/or analysed during the current study available from the corresponding author on reasonable request.

## Abbreviations

SMS: Self-management support
NCDs: Non-communicable diseases
PCA: Primary care academy
GOC: Goal-Oriented Care
IPC: Interprofessional Collaboration
SILCQ: Supporting, involving, listening, coordinating and questioning
COVID19: Coronavirus disease 2019
