# Supplementary material for: Self-management support (SMS) in primary care practice: a qualitative focus group study of care professionals’ experiences
Source: BMC Prim Care. 2024 Mar 1;25:76. doi: 10.1186/s12875-024-02317-4 (PMC10908026; doi:10.1186/s12875-024-02317-4)
Supplement: Supplementary file 1 — Supplementary Material 1. [file 12875_2024_2317_MOESM1_ESM.pdf]

Supplementary Table 1: precomposed interview guide questions on self-management support

**Wave 1**	• **How do you define and experience the concept of self-management/support? (Setting the stage)**<br>o Exploration of different domains<br>• **What specific strategies do you employ to support self-management in patients?**<br>• **List one competence that healthcare professionals need to develop to support self-management in patients.** (Repeat until saturation is reached)
**Wave 2**	• **What do you understand by the concept of self-management?**<br>**(Setting the stage)**<br>o Exploration of different domains<br>o Our research group defines self-management in individuals with chronic diseases as their ability to integrate their disease into their lives. Do you recognize your understanding of self-management in this definition?<br>• **What specific strategies do you employ to support self-management in practice?**<br>o Is this approach applicable to other target groups or patients?<br>o Barriers/facilitators?<br>• **How do you evaluate self-management?**<br>o How do you recognize whether it is successful or not?<br>o How do you adapt your support accordingly?<br>• **Discussion on the proposition: "Self-management is something you never do alone."**<br>o Agree or disagree? Explain.<br>• **List one competence that healthcare professionals need to develop to support self-management in patients.** (Repeat until saturation is reached)
**Wave 3**	There were no predefined questions. Findings from the two previous waves will be aligned with the panel to validate the data. Questions will be drafted after the results from the first waves are processed.
