# Supplementary material for: Self-management support (SMS) in primary care practice: a qualitative focus group study of care professionals’ experiences
Source: BMC Prim Care. 2024 Mar 1;25:76. doi: 10.1186/s12875-024-02317-4 (PMC10908026; doi:10.1186/s12875-024-02317-4)
Supplement: Supplementary file 2 — Supplementary Material 2. [file 12875_2024_2317_MOESM2_ESM.pdf]

Primary Care Academy

Membership of the Primary Care Academy: Roy Remmen[1], Emily Verté[1,2], Muhammed Mustafa Sirimsi[3], Peter Van Bogaert[4], Hans De Loof[5], Kris Van den Broeck[1], Sibyl Anthierens[1], Ine Huybrechts[1], Peter Raeymaeckers[6], Veerle Buffel[7], Dirk Devroey[2], Bert Aertgeerts[8], Birgitte Schoenmakers[8], Lotte Timmermans[8], Mieke Vermandere[8], Veerle Foulon[9], Anja Declerq[10], Nick Verhaeghe[11], Dominique Van de Velde[12,13], Pauline Boeckxstaens[14], An De Sutter[14], Patricia De Vriendt[12,13,15], Lies Lahousse[16], Peter Pype[14], Dagje Boeykens[12,14], Ann Van Hecke[14], Peter Decat[14], Rudi Roose[17], Sandra Martin[18], Anabel Wanzeele[18], Erica Rutten[18], Sam Pless[18], Vanessa Gauwe[13], Didier Reynaert[19], Leen Van Landschoot[20], Maja Lopez Hartmann[21], Tony Claeys[22], Hilde Vandenhoudt[23], Kristel De Vliegher[24], Susanne Op de Beeck[25].

1. Department of Primary Care and Interdisciplinary Care, Faculty of Medicine and Health Sciences. University of Antwerp. Antwerp. Belgium.
2. Department of Family Medicine and Chronic Care, Faculty of Medicine and Pharmacy. Vrije Universiteit Brussel. Brussel. Belgium.
3. Centre for research and innovation in care, Faculty of Medicine and Health Sciences. University of Antwerp. Antwerp. Belgium.
4. Workforce Management and Outcomes Research in Care, Faculty of Medicine and Health Sciences. University of Antwerp. Belgium.
5. Laboratory of Physio pharmacology, Faculty of Pharmaceutical Biomedical and Veterinary Sciences. University of Antwerp. Belgium.
6. Department of Sociology, Faculty of Social Sciences, Faculty of Social Sciences. University of Antwerp. Belgium.
7. Department of Sociology; centre for population, family and health, Faculty of Social Sciences. University of Antwerp. Belgium.
8. Academic Centre for General Practice, Faculty of Medicine. KU Leuven. Leuven, Department of Public Health and Primary Care, Faculty of Medicine, KU Leuven. Leuven. Belgium
9. Department of Pharmaceutical and Pharmacological Sciences, Faculty Pharmaceutical Sciences. KU Leuven. Leuven. Belgium.
10. LUCAS-Centre for Care Research and Consultancy, Faculty of Social Sciences. KU Leuven. Leuven. Belgium.
11. Research Group Social and Economic Policy and Social Inclusion, Research Institute for Work and Society. KU Leuven. Belgium.
12. Department of Rehabilitation Sciences, Occupational Therapy. Faculty of Medicine and Health Sciences. University of Ghent. Belgium.
13. Department of Occupational Therapy. Artevelde University of Applied Sciences. Ghent. Belgium.
14. Department of Public Health and Primary Care, Faculty of Medicine and Health sciences. University of Ghent. Belgium.
15. Frailty in Ageing (FRIA) Research Group, Department of Gerontology and Mental Health and Wellbeing (MENT) Research Group, Faculty of Medicine and Pharmacy, Vrije Universiteit, Brussels, Belgium.
16. Department of Bioanalysis, Faculty of Pharmaceutical Sciences, Ghent University. Ghent. Belgium.
17. Department of Social Work and Social Pedagogy, Faculty of Psychology and Educational Sciences. University Ghent. Belgium.
18. Expertise Centre Health Innovation. University College Leuven-Limburg. Leuven. Belgium.
19. E-QUAL, University College of Applied Sciences Ghent. Ghent. Belgium.
20. Department of Nursing, University of Applied Sciences Ghent. Ghent. Belgium.
21. Department of Welfare and Health, Karel de Grote University of Applied Sciences and Arts. Antwerp. Belgium.
22. LiveLab, VIVES University of Applied Sciences. Kortrijk. Belgium.
23. LiCalab, Thomas University of Applied Sciences. Turnhout. Belgium.
24. Department of Nursing–homecare, White-Yellow Cross. Brussels. Belgium
25. Flemish Patient Platform. Heverlee. Belgium.
